# Allometric Models for Predicting Aboveground Biomass and Carbon Stock of Tropical Perennial C_4_ Grasses in Hawaii

**DOI:** 10.3389/fpls.2017.00650

**Published:** 2017-05-02

**Authors:** Adel H. Youkhana, Richard M. Ogoshi, James R. Kiniry, Manyowa N. Meki, Mae H. Nakahata, Susan E. Crow

**Affiliations:** ^1^Department of Tropical Plant and Soil Sciences, University of Hawaii at Manoa, HonoluluHI, USA; ^2^Grassland Soil and Water Research Laboratory, United States Department of Agriculture, Agricultural Research Service, TempleTX, USA; ^3^Texas A&M AgriLife Research, Blackland Research and Extension Center, TempleTX, USA; ^4^Hawaiian Commercial & Sugar Company, PuuneneHI, USA; ^5^Department of Natural Resources and Environmental Management, University of Hawaii at Manoa, HonoluluHI, USA

**Keywords:** aboveground biomass, carbon sequestration, allometric models, C_4_ grasses, site-specific model, ratoon harvest

## Abstract

Biomass is a promising renewable energy option that provides a more environmentally sustainable alternative to fossil resources by reducing the net flux of greenhouse gasses to the atmosphere. Yet, allometric models that allow the prediction of aboveground biomass (AGB), biomass carbon (C) stock non-destructively have not yet been developed for tropical perennial C_4_ grasses currently under consideration as potential bioenergy feedstock in Hawaii and other subtropical and tropical locations. The objectives of this study were to develop optimal allometric relationships and site-specific models to predict AGB, biomass C stock of napiergrass, energycane, and sugarcane under cultivation practices for renewable energy and validate these site-specific models against independent data sets generated from sites with widely different environments. Several allometric models were developed for each species from data at a low elevation field on the island of Maui, Hawaii. A simple power model with stalk diameter (D) was best related to AGB and biomass C stock for napiergrass, energycane, and sugarcane, (*R*^2^ = 0.98, 0.96, and 0.97, respectively). The models were then tested against data collected from independent fields across an environmental gradient. For all crops, the models over-predicted AGB in plants with lower stalk D, but AGB was under-predicted in plants with higher stalk D. The models using stalk D were better for biomass prediction compared to dewlap H (Height from the base cut to most recently exposed leaf dewlap) models, which showed weak validation performance. Although stalk D model performed better, however, the mean square error (MSE)-systematic was ranged from 23 to 43 % of MSE for all crops. A strong relationship between model coefficient and rainfall was existed, although these were irrigated systems; suggesting a simple site-specific coefficient modulator for rainfall to reduce systematic errors in water-limited areas. These allometric equations provide a tool for farmers in the tropics to estimate perennial C_4_ grass biomass and C stock during decision-making for land management and as an environmental sustainability indicator within a renewable energy system.

## Introduction

The most effective biofuel feedstocks offer not only potential renewable energy to reduce our dependence on fossil fuels, but also minimization of net greenhouse gas flux during production (Lynd et al., 2008; DeLucia, 2016). Sustainably managed bioenergy cropping systems, including tropical perennial C_4_ grasses, can produce large amounts of biomass and increase soil sequestration (Matsuoka et al., 2014; Meki et al., 2014, 2015; Stokes et al., 2016). Perennial grasses can be grown by ratooning, a form of zero-tillage harvest that leaves roots and soil undisturbed, and can rapidly increase soil organic C while providing high biomass yields (Matsuoka and Stolf, 2012; Sumiyoshi et al., 2016). Many generalized models predict biomass and C stock in forestry and agroforestry systems (Nair et al., 2009; Chave et al., 2014; Vahedi et al., 2014; Ali et al., 2015; Kuyah and Rosenstock, 2015; Kuyah et al., 2016; Mganga, 2016), but only a few equations have been developed for non-forest crops (Navar et al., 2004; Nafus et al., 2009; Martin et al., 2013; Fard and Heshmati, 2014; Oliveras et al., 2014). However, Martin et al. (2013) used stalk base D, stalk H (the length from the base stalk to the base of the forth internode) to predict stalk biomass and soluble sugar concentration of Sweet Sorghum ^∗^(*Sorghum bicolor*) in Australia. Yield and growth rate data exist for multiple bioenergy crops across a range of environments in Hawaii and the tropics (Meki et al., 2014), yet to our knowledge there are no fully developed allometric models to predict AGB and C stock non-destructively for bioenergy crops.

Aboveground biomass and C stock can be determined by: (1) destructive methods, (2) remote sensing techniques (Saatchi et al., 2007; Asner, 2009; Chabi et al., 2016), and (3) allometric equations (Youkhana and Idol, 2011; Chave et al., 2014; Kuyah et al., 2016). Destructive methods are costly and time consuming compared to non-destructive methods (i.e., allometric models), while remote sensing is limited by access to technology and cloud cover and fly-over frequency (Xie et al., 2008). Therefore, the choice of an appropriate allometric model often is the most pragmatic, crucial step toward minimizing the errors and increasing the accuracy of AGB and C stock estimates (Chave et al., 2005; Molto et al., 2013). Allometric equations initially require an extensive destructive sampling. But, later the equations can be used as a non-destructive method to estimate AGB and C stock and, subsequently, to estimate the span of rotation, nutrient pools, and economic returns (Ares et al., 2002; Ekoungoulou et al., 2014; Cornet et al., 2015; Roxburgh et al., 2015). Developing new allometric models can improve the accuracy of biomass assessment protocols, and advance our understanding of architectural constraints on plant development (Chave et al., 2014). Allometric models are based on correlations between biomass and morphological characters, such as basal diameter (or area), height, canopy diameter, or canopy volume (Martin et al., 2013; Cornet et al., 2015; Kuyah et al., 2016). These parameters can be used individually, or combined in one allometric model (Brown, 1997, 2002).

No published work assesses allometries to predict AGB and C stock for cultivated C_4_ grasses, which have emerged as among the greatest potential crops for biofuel, in tropical and subtropical ecosystems. This lack provides an opportunity to develop site-specific allometric models that are more accurate than generalized models. Therefore, the objectives of the study were to: (1) develop allometric relationships and site-specific models to predict AGB and biomass C stock from measurements of stalk D or dewlap H of individual energycane, napiergrass and sugarcane stalks, (2) select best model based on goodness of fit indices, (3) test selected model against data sets generated from independent sites with different environments, and (4) assess the effect of environmental factors on model accuracy. To meet these objectives, three hypotheses were tested: (1) stalk D is the better predictor for biomass compared to dewlap H, (2) site specific models using stalk D and dewlap H are better predictors of biomass than generalized models, (3) climatic factor (s) will affect allometry pattern.

## Materials and Methods

### Study Site and Experimental Description

The study was conducted at the Hawaiian Commercial and Sugar (HC&S) plantation and Maui Agricultural Research Center (MARC) on the Island of Maui, Hawaii. The HC&S plantation is located in the central part of the Island of Maui and has practiced conventional sugarcane cultivation on 2-year rotation for over 100 years. In June–September 2011, four benchmark experimental plots that vary in elevation and soil type (**Table 1**) at HC&S were established in recently harvested sugarcane fields.

**Table 1:** Site information of field used to develop, calibrate and validate allometric model for biofuel crops on the island of Maui, Hawaii.

Field sites	Latitude	Longitude	Elevation (m)	Soil order	MAP (mm)	MAT (°C)	PET (mm)
718	20.854° N	-156.466° W	34	Mollisol	402.3	23.6	1368.8
609	20.897° N	-156.415° W	30	Oxisol	445.7	23.7	1613.3
410	20.830° N	-156.363° W	319	Aridisol	536.3	21.8	1365.2
Kula	20.756° N	-156.319° W	1025	Andisol	620.4	17.3	644.3

*MAP, mean annual precipitation; MAT, mean annual temperature; PET, permeant evapotranspiration, based on Giambelluca et al. (2013)*.

Three species: sugarcane (*Saccharum officinarum* cv. HA 65-7052), energycane (*S. officinarum* × *S. rubustom* cv. MOL-6081) and napiergrass hybrid (*Pennisetum purpureum* × *Pennisetum glaucum* cv. banagrass), were selected for their high potential for biomass production. The experimental life cycles of these crops are 2-year for sugarcane (current plantation practice), 1-year ratoon for energycane, and 6-month ratoon for the napiergrass hybrid. In Fields (F) 718 and 410, each plot (15 m × 11 m) consisted of four rows and F609, each plot (8.23 × 12.20 m) consisted of three rows of grass, with two lines per row. At the Kula site (MARC); each plot has similar dimensions as F609 except with shorter row length 4.6 m. Distances between rows and lines were 1.8 and 0.9 m, respectively. For all crops, 45 cm stem cuttings were planted end to end in 15 cm deep furrows in each line. Stem cuttings for F609 were planted in June, 2011, and F718 and F410 were planted in September, 2011. Plants were drip irrigated as needed to prevent stress. A total of 375 kg N ha^-1^ was applied to each field through the drip irrigation system as liquid urea. All plots received similar rates of fertilization. The field layout design was a randomized complete block design with three replicates.

The allometric models were initially developed on F718 and solar radiation, rainfall, air temperature, relative humidity and wind speed were recorded (**Table 2**). Napiergrass and energycane were harvested in September 2012. The napiergrass model was based on the first ratoon crop because of its presumed similarity to successive ratoon cycles. Sugarcane was harvested on September 2013. Data from two other HC&S fields (F410, F609) and the high elevation Kula site (MARC), which vary widely in climate and environmental conditions during the study period (**Table 3**), were used to validate and improve the models that were developed from F718 of all crops. The harvest cycles of all crops were repeated through 2015 in all fields for the validation and adjustment.

**Table 2:** Summary of weather data from site 718 during model develops for each crop^∗^.

Crop	Solar radiation	Air temperature	Rainfall	Relative Humidity	Wind speed
	(MJ/m^2^)	(°C)	(mm)	(%)	(km/hr)
Napiergrass	25.0	23.4	21.1	69	17.8
Energycane	20.7	22.7	157.2	71	16.1
Sugarcane	21.2	22.7	236.2	71	16.0

*^∗^All weather data in the table are average; except rainfall data is total accumulated, during the season of each crop cycle*.

**Table 3:** Summary of weather data at four sites on the island of Maui, Hawaii, collected during trails to validate allometric models^∗^.

Field	Solar radiation	Air temperature	Rainfall	Relative Humidity	Wind speed
	(MJ/m^2^)	(°C)	(mm)	(%)	(km/hr)
**Napiergrass**

718	22.8	24.5	169.9	74	15.7
609	19.8	22.2	485.4	75	14.5
410	22.5	23.4	305.1	75	9.7
Kula	16.0	19.1	426.0	80	4.4
**Energycane**
718	19.6	23.7	542.5	75	15
609	21.2	23.6	613.4	73	16.5
410	20.4	22.6	683.8	75	10.1
Kula	14.7	18.1	859.8	78	4.8
**Sugarcane**
718	19.6	23.6	1113.8	75	13.8
609	20.2	23.6	1027.4	75	13.4
410	19.6	22.9	1285.0	75	9.9
Kula	15.1	18.2	1585.7	78	5.2

*^∗^All weather data in the table are averages, except rainfall data was total accumulated during the cycle season of each crop*.

### Predicting Site-Specific AGB and C Stock

Thirty random stalks of each crop (10 from each rep) in F718 were destructively harvested for the development of allometric models. Basal D of the stalk was measured at 20 cm above the soil level, where the stalk was cut. Dewlap H was measured from the base cut to most recently exposed leaf dewlap. Each stalk (shoots and leaves) was weighed, and dried at 60°C until the constant weight was achieved. Biomass was regressed on stalk D or dewlap H to develop the site-specific models (D) and (H).

The site-specific models were compared to three published generalized equations for predicting tree and shrub biomass of tropical species (Brown, 1997; Sampaio and Silva, 2005; Brewbaker, 2008) because there are no models developed for C_4_ perennial biofuel grasses. Models were compared by estimating goodness of fit indices from the regression of biomass on stalk D, dewlap H. The Brown (1997) model require only stem D to predict total AGB for each plant; whereas, the model of Sampaio and Silva (2005) predicts total AGB based on D and H combined. The allometric equation developed for AGB of hybrid *Leucaena*-KX_2_ was included (Brewbaker, 2008). This model used canopy H alone as the predictor variable. Regression equations were analyzed using the PROC REG procedure in SAS version 9.3 (SAS Institute, 2007). For comparison and selection of allometric equations, we used goodness of fit measures, including *P*-values, the coefficient of determination (*R*^2^), the residual mean square (RMS), the Akaike Information Criterion (AIC), the bias-corrected AIC (AICc) tests (Burnham and Anderson, 2002; Ritter and Munoz-Carpena, 2013). The coefficient of determination for each model was calculated as *R*^2^ = (1-SSR)/corrected SST (Litton and Kauffman, 2008), The better models were selected as having the highest *R*^2^, and the lowest *P*-value, RMS, AIC, and AICc of biomass across the range of stalk D and dewlap H.

Carbon content of AGB was analyzed by oxidation and combustion using an elemental analyzer (Costech ECS4010) and C stock was estimated multiplying AGB by C concentration. For ABG carbon stock, the predictive equations were developed in the same way as for biomass.

### Model Validation and Calibration to Environmental Factors

The two best-fit models were tested against observations at sites F718, F410, F609 and Kula, which differed in solar radiation, air temperature, and wind speed that decreased as elevation increased in 2015 (**Table 3**). Field 718 was considered an independent validation site as well, because weather conditions were different between model development and validation periods (**Tables 2**, **3**). Twenty-five stalks of each species from each of three replicates at F718, F609, F410, and Kula were collected from each site on September 2015 to test the selected model. Stalk D, dewlap H, and dry weight were measured as before. The predicted AGB values for all sites were compared to observed AGB (*n* = 300) in a 1:1 plot.

For validation of selected allometric models, observed versus predicted AGB for individual stalks was plotted. Slope, *y*-intercept, and *R*^2^ were calculated from linear regression of predicted on observed values. In addition, mean square error (MSE), MSE-systematic, MSE-unsystematic, index of agreement (Willmott, 1981), and model efficiency (Ritter and Munoz-Carpena, 2013) were calculated from the same data. Sigma Plot software, V. 10 (Systat. Software, Inc., San Jose, CA, USA) was used to determine the *R*^2^ value and for linear regression analyses.

Systematic errors in the model parameters a and b were manually calibrated by site to investigate causal factors of the error. First, parameter a was kept constant while parameter b was calibrated to achieve near 0 *y*-intercept and near 1 slope in the 1:1 plot of predicted vs. observed ABG. Second, parameter b was kept constant and parameter a was calibrated to achieve the same *y*-intercept and slope previously. Calibrated values for a and b were regressed against climatic factors (solar radiation, air temperature, rainfall, relative humidity, and wind speed) for each site and crop.

## Results

### Predicting AGB and C Stock

All of the generalized models evaluated were highly significant (*p* < 0.01), with some having high *R*^2^ (0.89), but none of them provided a better fit than the site-specific model D for each crop at F718 (**Table 4**). The simple power, site-specific model (D), was found to be the best predictor of AGB of individual plant of napiergrass, energycane, and sugarcane (*R*^2^ = 0.98, 0.96, and 0.97, respectively), with minimum RMS, AIC, and AICc (**Table 4**). Logarithmic transformation of the data did not reduce error variance with stalk D or improve the fit for AGB (data not shown). Dewlap H also predicted biomass well for napiergrass, energycane, and sugarcane (*R*^2^ = 0.93, 0.91, and 0.94, respectively), but not as well as the site-specific model D.

**Table 4:** Site specific and generalized allometric models for napiergrass, energycane and sugarcane and goodness of fit indices.

Reference	Model	*R*^2^	RMS	AIC	AICc	*P*-value
**Napiergrass**
Site specific model (D)	*Y* = 151.26 × D^0.68^	0.98	27	134	135	<0.01
Site specific model (H)	*Y* = 47.98 × H^0.35^	0.93	47	136	137	<0.01
Brown (1997)	*Y* = exp [-2.134+2.530 × ln(D)]	0.55	702	199	200	<0.01
Brewbaker (2008)	*Y* = 5.37 × l0^-5^ × H^2.714^	0.89	476	185	186	<0.01
Sampaio and Silva (2005)	*Y* = 0.0612 × (D × H)^1.5811^	0.89	316	171	177	<0.01
**Energycane**
Site specific model (D)	*Y* = 144.99 × D^0.72^	0.96	18	89	90	<0.01
Site specific model (H)	*Y* = 48.90 × H^0.36^	0.91	19	91	92	<0.01
Brown (1997)	*Y* = exp [-2.134+2.530 × ln(D)]	0.88	28	102	103	<0.01
Brewbaker (2008)	*Y* = 5.37 × l0^-5^ × H^2.714^	0.75	149	154	155	<0.01
Sampaio and Silva (2005)	*Y* = 0.0612 × (D × H)^1.5811^	0.72	1110	212	213	<0.01
**Sugarcane**
Site specific model (D)	*Y* = 309.34 × D^0.71^	0.97	491	187	188	<0.01
Site specific model (H)	*Y* = 16.08 × H^0.73^	0.94	895	205	206	<0.01
Brown (1997)	*Y* = exp [-2.134+2.530 × ln(D)]	0.68	47200	325	326	<0.01
Brewbaker (2008)	*Y* = 5.37 × 10^-5^ × H^2.714^	0.78	1950	295	296	<0.01
Sampaio and Silva (2005)	*Y* = 0.0612 × (D × H)^1.5811^	0.87	4920	256	257	<0.01

**FIGURE 1:**
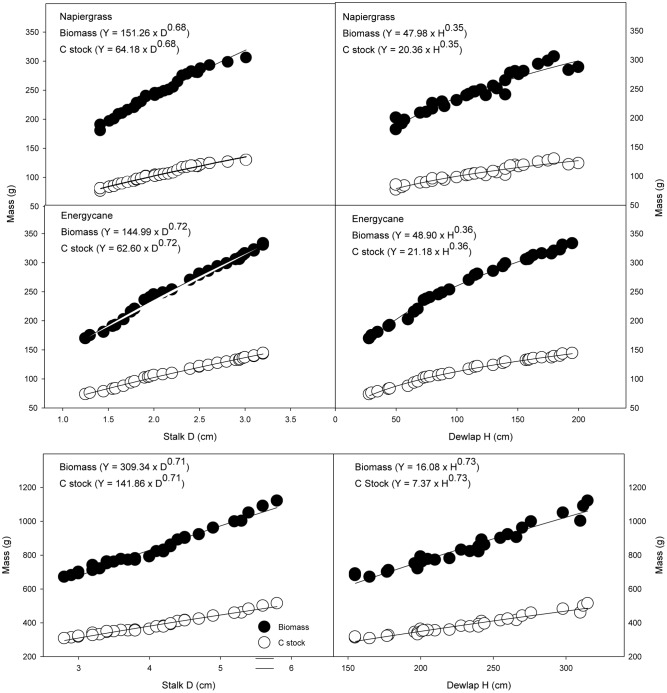
**Aboveground biomass and C stock as related to stalk D and dewlap H for napiergrass hybrid, energycane and sugarcane**. The power equations describing these relationships are the site specific models (D) and (H). Carbon stock for napiergrass hybrid, energycane and sugarcane are 42.4 and 43.0 and 45.6 % of biomass, respectively.

As with AGB prediction and because C stock is directly related to biomass quantification, the simple power model using only stalk D as a single independent variable was found to be the best predictor of C stock of individual plant of all crops (**Figure 1**).

### Validation, Calibration, and Adjustment of AGB Models

**FIGURE 2:**
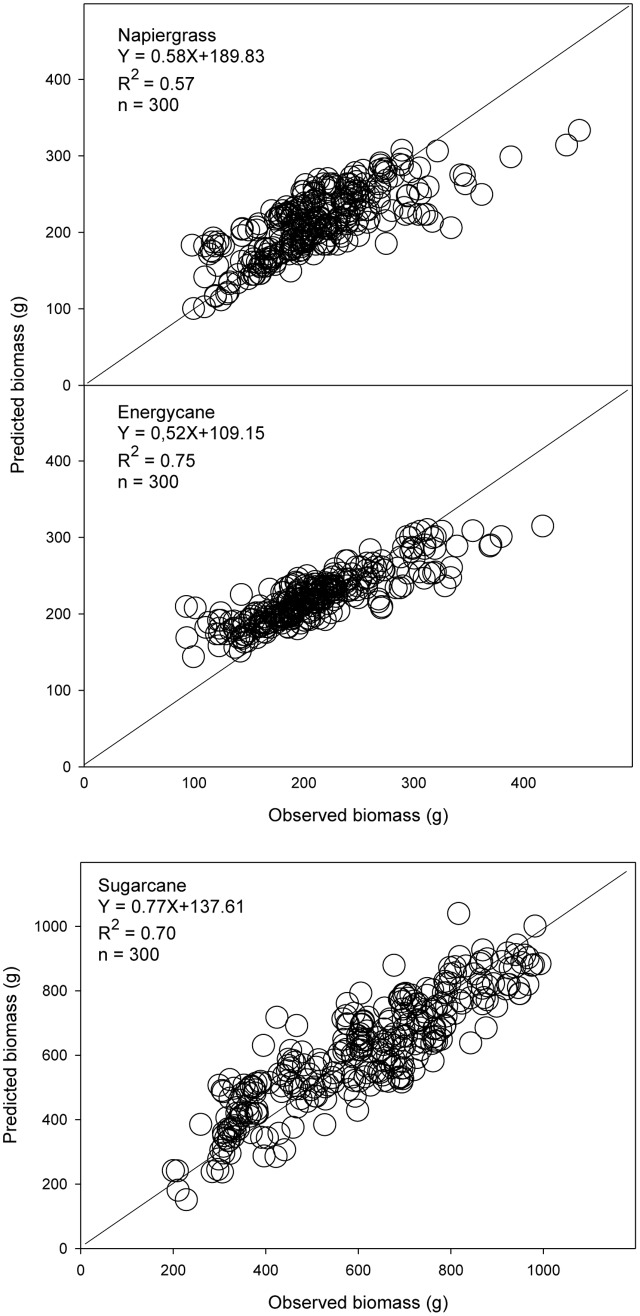
**Observed and predicted AGB from independent sites (*n* = 300) for napiergrass, energycane and sugarcane on the island of Maui, Hawaii, where stalk D is a predictor**. The solid line is the 1:1 line. Linear regression equation and associated *R*^2^ are shown.

The models using stalk D were better for biomass prediction compared to dewlap H models, which showed weak validation performance, in F718, F609, F410, and Kula for napiergrass, energycane, and sugarcane (**Table 5**). Regression analysis of predicted on observed AGB showed *y*-intercept closer to 0 and lower MSE for stalk D than dewlap H. In addition, both index of agreement and model efficiency were closer to 1 for stalk D than dewlap H (**Figure 2** and **Table 5**-validation part). Although stalk D model performed better, MSE-systematic was ranged from 23 to 43% of MSE for all crops.

**Table 5:** Statistics of validated and rainfall-adjusted site specific models for napiergrass, energycane and sugarcane at independent sites using stalk D and dewlap H as predictors for validation and stalk D as predictor for rainfall-adjustment models.

	Napiergrass		Sugarcane		Energycane	
	Stalk D	Dewlap H	Stalk D	Dewlap H	Stalk D	Dewlap H
**Testing Validation site specific models**
Slope	0.58	0.12	0.77	0.44	0.52	0.31
Intercept	189.83	351.06	137.61	603.26	109.15	311.58
*R*^2^	0.57	0.22	0.70	0.18	0.75	0.29
d-Index agreement	0.86	0.21	0.94	0.42	0.92	0.26
Model efficiency	0.59	-8.97	0.78	-9.57	0.66	-8.69
MSE	1238	12214	8340	9240	775	2873
MSE systematic	512	514	1890	5040	336	2170
MSE unsystematic	726	11700	6450	4200	439	703
**Testing rainfall-adjusted site specific models**
Slope	0.89		0.89		1.01	
Intercept	-5.06		34.68		-10.35	
*R*^2^	0.69		0.84		0.87	
d-Index agreement	0.99		0.95		0.99	
Model efficiency	0.90		0.81		0.99	
MSE	2703.50		8249.14		1411.81	
MSE systematic	500.58		1709.18		378.59	
MSE unsystematic	2202.92		6539.96		1033.22	

**FIGURE 3:**
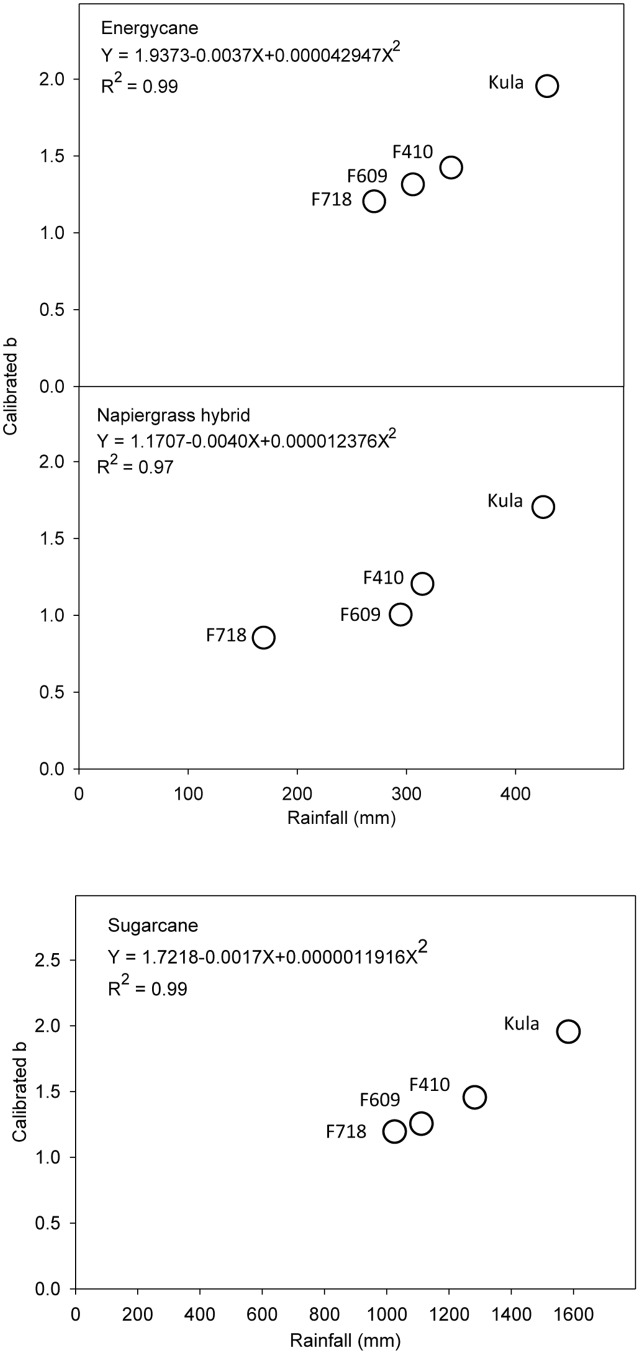
**The effect of rainfall on parameter b of site specific model (D) for energycane, napiergrass, and sugarcane**. Parameter b was adjusted to closely match predicted to observed AGB at each of the four sites (F718, F609, F410, and Kula).

Among the environmental parameters, rainfall was the greatest factor causing systematic error for the site-specific model (D). Parameter b was better to meet the objective of slope to be equal to 1 and *y*-intercept equal to 0 for all crops than parameter a (data not shown). So, calibrated values of parameter b were plotted against weather parameters from each site. By visual inspection, parameter b seems to be highly and strong related to rainfall (*R*^2^ more than 0.95 for all crops) (**Figure 3**). However, after b-rainfall adjustment and testing with independent data, the site specific models were more robust to improve the prediction of ABG biomass for all crops compared to validation stage (**Table 5**).

## Discussion

Relationships among plant parameters often provide an effective means to estimate AGB and thus C stock (Brown, 2002; Chave et al., 2005; Northup et al., 2005; Youkhana and Idol, 2011; Martin et al., 2013; Mganga, 2016), which can extend to belowground C pools using common inventory variables (Youkhana and Idol, 2011). However, to minimize bias, the development of locally derived diameter-height relationships is advised whenever possible (Feldspauch et al., 2011; Martin et al., 2013). We found that stalk D and dewlap H were highly related to AGB and C stock, using a simple power allometric equation. As expected, stalk D was sufficient for predicting AGB and biomass C stock. Similarly, stem diameter alone was observed to be a reliable predictor of total biomass and C stock for a variety of species and ecosystems (Sampaio and Silva, 2005; Segura and Kanninen, 2005; Litton and Kauffman, 2008; Beets et al., 2012; Kuyah and Rosenstock, 2015; Roxburgh et al., 2015). Furthermore, diameter at breast height (DBH) often is used to predict AGB for tropical trees and shrubs (Fownes and Harrington, 1992; Chave et al., 2014; Ali et al., 2015; Kuyah et al., 2016). Claesson et al. (2001) argued that using only one response variable in allometric models to estimate biomass was less accurate, so, we also used dewlap H as an alternative predictor variable. However, the combination of stalk D and dewlap H did not improve the biomass prediction.

**FIGURE 4:**
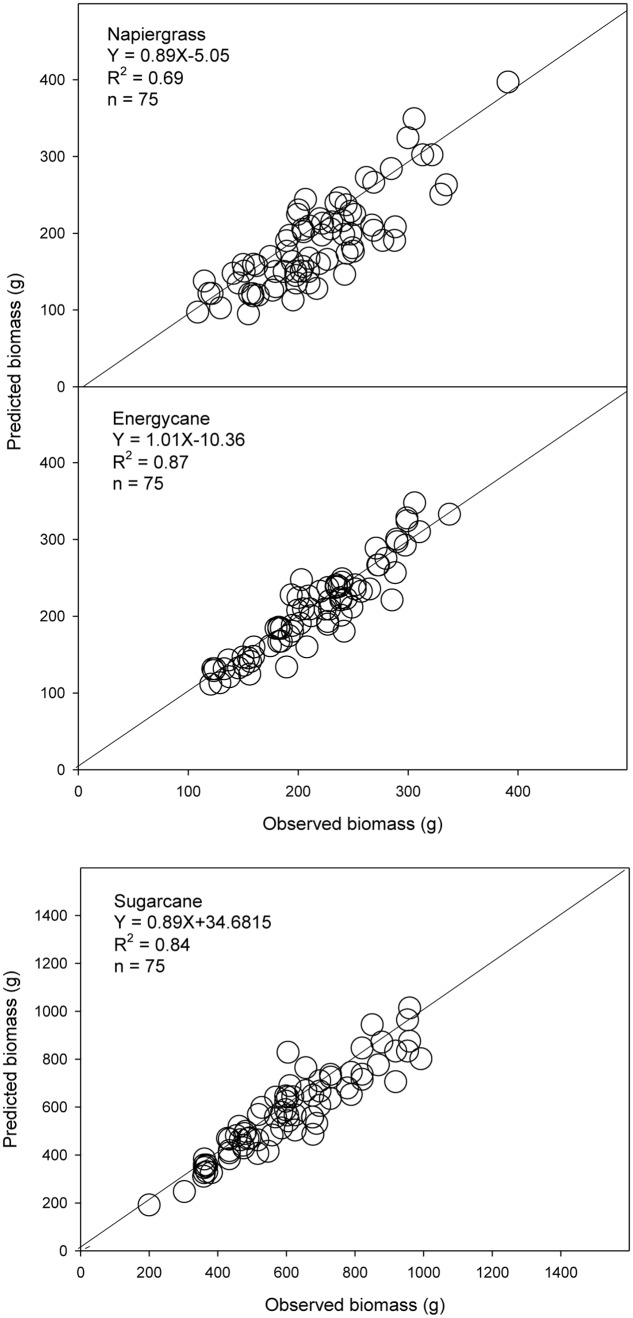
**Observed and predicted AGB from independent data using adjusted b-rainfall models for napiergrass hybrid, energycane and sugarcane**. The solid line is the 1:1 line. Linear regression equation and associated *R*^2^ are shown.

Here, the site-specific model (D) was the best predictor of AGB for all crops in lower rainfall sites; however, the prediction was poor at high rainfall sites and this might be due to rainfall impact on growth and morphology of such C_4_ grasses across the elevations. Also, using drip irrigation and fertigation systems in these sites, have caused a limitation on root zone around the plant (unpublished data), and rainfall may increase this root zone and impact the AGB growth. Our results suggest a flexible allometry (i.e., change in architecture) that allocates more biomass to aboveground as rainfall increases. Similarly, decreased biomass allocation to root and increased allocation to shoot due to higher soil moisture and low light conditions has been observed in forest trees (Niinemets, 2010; Schall et al., 2012). The rainfall calibration allowed applying the model to each site based on rainfall-modifier of each crop cycle, which leads to improving the prediction (**Figure 4** and **Table 5**).

The results presented in this study show the potential of predicting AGB and C of individual stalk; however, the yield has not been estimated in this study. To predict biomass per unit area, the number of stalks per unit area is needed, and this needs further investigation. The number of stalks per unit area may be based relationships such as those found by Kikuzawa (1999) for forest trees.

## Conclusion

To our knowledge, this is the first attempt to develop site-specific allometric models for napiergrass, energycane and sugarcane cultivated for biofuel production. The allometric equations in this paper represent a new tool for the practical evaluation of management and a non-destructive estimation of biomass for biofuel feedstock production in Hawaii and other tropical regions. However, changing environmental conditions over region or time may influence the allometric relation between the predictor variable and biomass. In the present case, parameter b was highly related to rainfall. This suggests that adjusting parameter b according to the rainfall at a particular site or time of any crop cycle, will make the model more robust.

## Author Contributions

Resources were provided by Hawaiian Commercial and Sugar (HC&S) in terms of (1) Field site location and land availability within HC&S plantation. (2) Cost-sharing to meet the requirements of one funding source through use of space within the headquarters and processing facility for office and laboratory needs. As the primary stakeholder for the project, MN assisted with funding acquisition by developing budgets to cover the costs of the HC&S field crew for farming and irrigation. AY, RO, SC, JK, MM: conceptualization; AY, RO, SC: methodology; AY, RO, SC: validation; AY, RO: formal analysis; AY, RO, SC, JK, MM: investigation; SC, RO, JK, MN: resources; AY, RO: data curation; AY, RO: writing (original draft preparation); AY, RO, SC, JK, MM: writing (review and editing); AY, RO, SC: visualization; AY, RO, SC: supervision; SC, JK, RO: project administration; and SC, JK, RO, MN: funding acquisition.

## Conflict of Interest Statement

The authors declare that the research was conducted in the absence of any commercial or financial relationships that could be construed as a potential conflict of interest.
